# Plant growth-promoting rhizobacterial inoculation to improve growth, yield, and grain nutrient uptake of teff varieties

**DOI:** 10.3389/fmicb.2022.896770

**Published:** 2022-10-21

**Authors:** Zerihun Tsegaye, Tesfaye Alemu, Feleke Adey Desta, Fassil Assefa

**Affiliations:** ^1^Department of Microbial, Cellular and Molecular Biology, College of Natural and Computational Sciences, Addis Ababa University, Addis Ababa, Ethiopia; ^2^Microbial Biodiversity Directorate, Ethiopian Biodiversity Institute, Addis Ababa, Ethiopia

**Keywords:** agronomy, biodiversity, fertilizer, inoculant, treatment, seed, stress

## Abstract

Inoculation of plant growth-promoting rhizobacteria (PGPR) improves the growth, yield, and plant nutrient uptake, as well as rhizosphere fertility, without harming the environment and human health. This study aimed to examine the effect of either individual or consortium of PGP bacterial inoculation on the growth, yield, and grain nutrient uptake of teff varieties. Three potential PGPR strains (i.e., *Pseudomonas fluorescens biotype G*, *Enterobacter cloacae ss disolvens*, and *Serratia marcescens ss marcescens*) were used for this study. Field evaluation was carried out in RCBD with 5 treatments. Highly significant (*P* < 0.001) differences were observed among treatments for plant height (PH), panicle length (PL), number of the total spike (NTS), shoot dry weight (SDW), grain yield (GY), and straw yield (SY). There was also teff variety that significantly (*P* < 0.01) affects PL, SDW, and SY. However, the interaction effect of the two factors (treatment*variety) did not significantly influence teff agronomic traits and grain nutrient uptake. The highest PH (133.5 cm), PL (53.2), NTS (30.9), SDW (18.1 t/ha), SY (10.7 t/ha), and GY (2.7 t/ha) were observed on Dukem variety (Dz-01-974) inoculated with PGPR consortium. Wherein 2.2 fold increase was observed in grain yield per hectare over the control. Inoculation of PGPR consortium showed better performance in promoting plant growth, yield, and grain nutrient uptake of teff varieties compared with the individual PGP bacterial application, and PGPR consortium could be used as inoculants to enhance teff production and productivity.

## Introduction

Teff [*Eragrostis tef* (Zucc.) Trotter] is an indigenous tropical cereal crop of Ethiopia, and the country is the center of origin and diversity for the crop (Vavilov, 1951). Its grain is used to make injera, a delicious traditional fermented pancake, which is one of the major staple foods for about 70 million inhabitants (60% of the entire population) (Reda, 2015). Teff grain has an excellent nutritional profile, with high dietary fiber and high levels of minerals, proteins, and carbohydrates (Baye, 2014). Doris (2002) reported that teff contains 11% protein and is an excellent source of essential amino acids. It has a low glycemic index; it is free from gluten and serves as an alternative food source for people with type 2 diabetes and celiac disease (Baye, 2014).

In Ethiopia, about 6.5 million smallholder farmers grow teff, which is equivalent to 30% of the total area allocated to cereals followed by maize (23%), sorghum (18%), and wheat (17%) (Central Statistics Agency [CSA], 2019). In the 2019/2020 cropping season, the total area covered with teff was 3.1 million hectares with a production of 5.74 million tons. The average productivity of teff in Ethiopia is very low (1.85 t/h) at the smallholder farmer level. The main reason for low teff productivity is nutrient deficiency, susceptibility to lodging (Habtegebrial et al., 2007), low genetic yield potential (Haileselassie et al., 2016), and drought, particularly in the low altitude.

Currently in Ethiopia, teff production and productivity improvement practices were dependent on the heavy application of chemical inputs (such as fertilizers, pesticides, and herbicides), which may have a deleterious effect on soil fertility and nutritional value of farm products. Excessive use of those chemical inputs causes environmental pollution, which together has a major impact on human and animal health through an accumulation of heavy metals and other toxic substances (Tchounwou et al., 2012).

Chemical fertilizers contain acid radicals, like hydrochloride and sulfuric radicals, and hence increase the soil acidity and adversely affect biological diversity within the agricultural land. Some plants also can absorb recalcitrant compounds from the contaminated soil and cause systematic disorders in the consumers (Alori and Babalola, 2018). Therefore, the increasing awareness of environmental pollution and product contamination due to indiscriminate use of chemical inputs has led to the search for new biological technology to improve crop productivity and grain quality without threatening consumers’ health. Either application of either individual or a consortium of plant growth promoting bacteria (PGPR), which can act as a biofertilizer and biocontrol agent, is one of the alternative mechanisms to use hazardous chemical fertilizer (Tobergte and Curtis, 2013). They are environmental-friendly and renewably provide nutrients to maintain soil health and biology without affecting the environment and human health.

PGPR inoculants constitutes a biological tool to enhance plant nutrition and mitigate the negative impact of conventional chemical inputs. *Pseudomonas, Bacillus, Azospirillum, Azotobacter, Enterobacter*, and *Serratia* are the main genera of PGPR that enhance crop productivity and grain quality (Ferreira et al., 2019). PGPR application can increase plant growth, yield, yield components, and grain nutrient uptake by improving the availability of the essential nutrients, growth hormones, production of different lytic enzymes, and secondary metabolites, which inhibit plant pathogens (Gopalakrishnan et al., 2015). According to Zewdie et al. (2000), inoculation of teff varieties with indigenous *Azospirillum* isolates significantly increased grain yield (GY) up to 12% over control.

The synergy of two or more PGPR inoculants has been investigated in the last two decades after simultaneously inoculated in the same plant (Mpanga et al., 2019). Several researchers reported that inoculation with consortia of PGPR has better plant growth promotion as compared with individual inoculations because the individual strain is supplementing each other for their beneficial traits (Singh et al., 2014). According to Wang et al.’s (2020) report, the application of PGPR consortia can increase the production and growth of maize and cucumber plants compared with the inoculation of individual bacteria. Moreover, Souza et al. (2015) reported that plant inoculation with a consortium of several PGPR strains increases plant growth and yield than that with the individual strains, likely reflecting the various mechanisms used by each strain in the consortium. Despite the various plant growth promotion and biocontrol benefits associated with PGPR listed in the literature, research conducted to examine the effect of native PGPR application on teff to improve growth, yield, and grain nutrient uptake is limited. This study aimed to examine the effect of individual or consortium native PGPR inoculation on the growth, yield, and yield-related traits as well as grain nutrient uptake of teff varieties under field conditions.

## Materials and methods

### Description of the study area

The study was conducted at the Debrezeit Agricultural Research Center (DZARC) during the 2019 main cropping season. The research site is geographically located at 08° 44′N and 38°58′E and has an altitude of 1,900 m.a.s.l. DZARC is located 47 km southeast of Addis Ababa. The mean long-term annual rainfall recorded at the station is 660 mm, and the average annual minimum and maximum temperatures are 12 and 27.4°C, respectively (Tesfahun, 2018). The texture of the soil in the experimental site was silt loam and composed of 14% clay, 32% sand, 54% silt, and 1.26% organic carbon content, which is considered to be low (Roy et al., 2006). According to Olsen et al.’s (1954) phosphorus (P) rating (m/kg), the available P content of the experimental site soil (<3) is low. The pH of the soil was 6.96, which is within the suitable range (4–8) for teff production, and the total nitrogen (N) of the soil (0.12%) is medium, as rated by Havlin et al. (1999).

### Materials used in the experiment

Two teff varieties named Magna (Dz-01-196) and Dukem (Dz-01-974) were obtained from DZARC. Teff varieties were selected based on consumer and farmer’s preferences for injera making quality and market demand, respectively. Three potential PGPR strains (i.e., *Pseudomonas fluorescens biotype G*, *Enterobacter cloacae ss disolvens*, and *Serratia marcescens ss marcescens*) identified from teff varieties in the previous study were used as inoculants (Tsegaye et al., 2021). The three PGPR strains were selected from 65 potential identified PGPR, based on different plant growth-promoting (PGP) traits such as plant growth and yield-enhancing properties, biotic and abiotic stress tolerance property, in addition to seed germination capability during laboratory and greenhouse evaluation. Detailed information on the bacteria used for this study is given in Table 1.

**TABLE 1:** Potential PGPR strains were selected for the field experimental trail.

Code of bacterial isolates	PGP properties			Biocontrol properties			Abiotic stress tolerance properties			Seed germination status
	PS	IAA	NF	Pro	HCN	EPS	SL	pH	TP	SVI
*Serretia marcescens ss marcescens*	+++	+++	+	+++	+	++	5	4, 5, 7, and 9	40	530
*Pseudomonas fluorescent biotype G*	+++	+++	++	++	+++	+++	10	5, 7, and 9	30	470
*Enterobacter cloacae ss disolvens*	++++	++	+++	+++	+	+++	15	5, 7	40	540

Tsegaye et al. (2021).

PS, phosphate solubilization; IAA, indole acetic acid; NF, nitrogen fixation; EPS, exopolysaccharide; SL, salinity; TP, temperature; SVI, seed vigor index; +, low; ++, moderate; +++, high; and ++++, extremely high.

### Plant growth-promoting rhizobacteria strains compatibility test

Compatibility among the three selected PGPR strains was tested to formulate bacterial consortia. The method described by Nikam et al. (2007) with slight modifications was used for *in vitro* bacterial compatibility testing. PGPR cultures were streaked on nutrient agar plates in such a way that for every single bacterial culture in the center of the plate, other cultures were streaked radiating from the center. The plates were incubated at 30°C for 48 h. The zone of inhibition was observed and recorded. Bacterial strains that do not show a zone of inhibition on the growth medium indicate the compatibility of the strains.

### Seed surface sterilization and bacterial inoculant preparation

Teff seeds were surface sterilized with 70% alcohol for 3 min, followed with 1% hypochlorite for 5 min, and rinsed 5 times with sterile distilled water (Bello et al., 2018). PGPR inoculant was prepared using the bacterial seed coating method. Seed coating is a technique in which an active ingredient (e.g., bacterial inoculant) is applied to the surface of the seed with the help of a binder (adhesive) substance (Rocha et al., 2019). For this, a nutrient broth medium amended with 1% carboxyl methylcellulose (CMC) was prepared. Individual or consortium of PGPR inoculants with a concentration of 10^8^ CFU/ml were transferred into the prepared medium. Surface sterilized seeds of two teff varieties were immersed in a medium containing the suspensions of PGPR either alone or in a consortium and incubated for 1 h using shaker incubator. Then, the bacterial seed coating was completed.

### The experimental design and treatments

The treatment of the field experiment consists of three potential PGPR, either individual or in consortium form. Five treatments were established for the experiment, namely, T1-(PGPR1), T2-(PGPR2), T3-PGPR3), T4-(PGPR1 + PGPR2 + PGPR3), and T0-Control treatment (non-inoculated seeds). The experiment was laid out in a randomized complete block design (RCBD).

### The experimental procedure

The land was prepared by tractor plowing, and the seedbeds were leveled and compacted. A plot size of 2 m × 2 m (4 m^2^) with 20 cm row spacing and a total of 30 plots were used. The spacing between plots and blocks was 0.5 and 1 m, respectively. For inoculum preparation, selected potential PGPR were grown in a nutrient agar medium at 30°C. The cells grown in the exponential phase were harvested by centrifugation (at 10,000 rpm for 5 min), and then, the pellet was washed two times to obtain pure cells. For inoculum preparation, the pelleted cells were resuspended into the nutrient broth and adjusted to a final concentration of 10^8^ CFU/ml) used for each treatment. Surface sterilized seeds of two teff varieties were immersed in the inoculated broth and incubated at 30°C for 1 h in each of the individual bacterial suspension or a consortium of the three strains. The inoculated seeds were transferred to sterilized filter paper and allowed to air-dry in a laminar flow hood (Leyva-Madrigal et al., 2015). Inoculated seeds of two varieties were hand drilled at the rate of 5 kg per hectare, i.e., 2 g/plot.

Fifteen days after teff seedling emergence, a second bacterial inoculation was performed, in which 5 ml of bacterial inoculums (10^8^ CFU/ml) was added per plot. In addition, 30 days after teff seedling emergence, a third bacterial inoculation was performed at the same concentration as used previously. Plots were kept free of weed by hand weeding without using herbicides.

### Field data collection and measurement

At the physiological maturity, plant growth, yield, and yield-related data were collected before and after harvesting according to the teff descriptor lists (Seyfu, 1993). Ten plants were selected from the central two rows of each plot to measure plant growth and growth-related parameters. Harvesting was done manually using a hand sickle from an area of 1.8 m × 1.8 m (3.24 m^2^) to measure grain yield (GY), straw yield (SY), and yield-related parameters. In addition, lodging index (LI) shows that the level of lodging was measured just before the time of harvest by visual observation. It was determined by the angle of inclination of the main stem from the vertical line to the base of the stem measured on a 1–5 scale, where 1 (0–15°) indicates no lodging, 2 (15–30°) indicates 25% lodging, 3 (30–45°) indicates 50% lodging, 4 (45–60°) indicates 75% lodging, and 5 (60–90°) indicates 100% lodging (Donald, 2004). Data recorded on lodging percentage were subjected to arc sign transformation described for percentage data by Gomez and Gomez (1984).

### Teff grain nutrient contents determination

The following macro- and micronutrients such as nitrogen (N), phosphorus (P), sulfur (S), potassium (K), calcium (Ca), magnesium (Mg), zinc (Zn), and iron (Fe) were determined using standard procedure (Jull et al., 2018). Teff seeds were oven-dried at 60°C for 48 h and milled. For each treatment, a 1,000 mg of milled grain was used to determine grain nutrient contents. The N concentration was determined by means of complete digestion in concentrated H_2_SO_4_ and subsequent distillation using the micro-Kjeldahl method. Total K and P were determined by using a flame photometer and metavanadate colorimetry, respectively. Total Ca, Mg, and Zn contents in grain were determined using an inductively coupled plasma–atomic emission spectrometer. The protein content was quantified by Kjeldahl’s method, and the samples were read on a UV-VIS spectrophotometer. The analysis was developed according to the methodology described by Miyazawa et al. (1999).

### Data analysis

All collected data were analyzed using the R software version 3.6 statistical analysis system following the appropriate procedures of RCBD. A two-way ANOVA was conducted to test the significance level of the variables at P ≤ 0.05. A comparison of the individual treatment means was performed using the least significant difference (LSD).

## Results

### Effect of PGPR inoculation on teff agronomic traits

Analysis of variance for agronomic traits showed that traits like plant height (PH), panicle length (PL), the number of total spikelets, shoot dry weight (SDW), grain yield, straw yield, and harvest index were significantly affected by PGPR inoculants at 0.1% probability level, while lodging index was significantly affected at 5% probability level by teff variety. On the contrary, the interaction effect of variety and treatment did not significantly affect the agronomic traits of the two varieties (Table 2).

**TABLE 2:** Probability values for treatment, variety, variety and treatment interaction effects on growth, yield and related traits of teff.

S.O.V	DF	Growth, yield, and yield-related traits								
		PH	PL	NTS	NFT	SDW	GY	SY	HI	LI
TM	4	2129.5**	290.2**	120**	24.8*^NS^*	11.6**	0.31**	3.4**	0.01**	0.78*^NS^*
VT	1	240.8*	264.0**	41.3*^NS^*	3.7*^NS^*	5.4**	0.06*^NS^*	2.5**	0.001	20.8*
TM*VT	4	2.6*^NS^*	9.3*^NS^*	2.5*^NS^*	6.6*^NS^*	0.36*^NS^*	0.03*^NS^*	0.23*^NS^*	0.001*^NS^*	3.1*^NS^*
Error	20	45.2	9.3	10.9	9.5	0.30	0.009	0.25	0.001	4.20

S.O.V, source of variation; DF, degree of freedom; TM, treatment; VT, variety; PH, plant height; P, panicle length; NTS, number of total spikelet; NFT, number of fertile tiller; SDW, shoot dry weight; GY, grain yield; SY, straw yield; HI, harvest index; LI, lodging.

*, **, and ***: statistically significant at *P* ≤ 0.05, *P* ≤ 0.01, and *P* ≤ 0.001 probability levels, respectively; NS, not significant.

#### Effect of PGPR inoculation on teff varieties growth and growth-related traits

Teff varieties inoculated with individual or consortium PGPR showed significantly (*P* < 0.001) increased PH compared with control (Table 3). The longest PH (133.5 cm) was observed on Dz-01-974 inoculated with the consortium of the PGPR, and the shortest PH (84.1 cm) was observed on uninoculated Dz-01-196. Similarly, the PL of both varieties was significantly (*P* < 0.001) increased by inoculation of either individual or consortium PGPR. The longest PL (53.2 cm) was observed on Dz-01-974 inoculated with the bacterial consortium, and the shortest PL (31.7 cm) was observed on uninoculated Dz-01-196, which increased 1.59 folds over control. The number of total spikelets of Dz-01-196 was significantly (*P* < 0.01) affected by the inoculation of either individual or consortium PGPR. The smallest NTS (18.4) was observed on uninoculated Dz-01-196, and the highest NTS (30.9) was recorded on Dz-01-974 inoculated with PGPR consortium, which exceeds 1.58 folds over the control. The number of fertile tillers was significantly affected by *E. cloacae ss dissolvens* inoculation. The maximum NFT (12.3) was observed on Dz-01-974, and the shortest PL (5.1) was recorded on uninoculated Dz-01-196 (Table 3).

**TABLE 3:** PGPR inoculation effects on teff varieties growth and growth-related traits.

Treatment	Mean teff growth-promoting traits							

	PH		PL		NTS		N FT	
				
	M	D	M	D	M	D	M	D
Control	84.1^b^	88.8^b^	31.7^b^	33.5^b^	18.4^b^	19.5^b^	5.1^a^	5.7^b^
*S. marcescens ss marcescens*	122.5^a^	128.9^a^	44.3a	50.1^a^	27.9^a^	29.7^a^	9.7^a^	8.6a^b^
*P. fluorescens biotype G*	124.8^a^	129.6^a^	43.6^a^	51.5^a^	26.1^a^	30.7^a^	6.9^a^	7.4^ab^
*E. cloacae ss dissolvens*	125.5a	133.1^a^	43.0^a^	50.9^a^	27.7^a^	29.6^a^	8.0^a^	12.3^a^
PGPR consortium	128.7^a^	133.5^a^	46.7^a^	53.2^a^	28.6^a^	30.9^a^	9.8^a^	10.3^ab^
LSD (5%)	8.35	13.49	5.94	5.11	6.02	6.04	4.24	6.71
*P*-value	0.001	0.001	0.001	0.001	0.01	0.01	0.23	0.24

PH, plant height; PL, panicle length; NTS, number of total spikelet; NFT, number of the fertile tiller.

Different letters indicate significant differences at *P* ≤ 0.05 according to the LSD test.

#### Effect of PGPR inoculation on teff yield and yield-related traits

Individual treatment means comparison results showed that the SDW, GY, and SY of both varieties were significantly (*P* < 0.001) influenced by inoculation of PGPR inoculants either alone or in combination (Table 4). The maximum SDW (18.1 t/ha) was obtained from Dz-01-974 inoculated with the PGPR consortium, and the minimum SDW (5.8 t/ha) was obtained from uninoculated Dz-01-196. The consortium inoculation which increased 3.1 folds of SDW over control. Regarding the GY, the maximum GY (2.7 t/ha) was obtained from Dz-01-974 inoculated with the PGPR consortium, and the minimum GY (0.86 t/ha) was recorded from uninoculated Dz-01-196. The magnitude of increase in GY was higher by about 450% over the uninoculated plots. Similarly, the lowest SY (3.5 t/ha) was obtained from uninoculated Dz-01-196, and the highest SY (10.7 t/ha) was obtained from Dz-01-974 inoculated with the PGPR consortium, which increased 2.2 folds over control plots. The results of the individual treatment mean comparison revealed that the harvest index of both varieties was significantly (*P* < 0.05) influenced by the application of either individual or consortium PGPR. The minimum HI (16%) was observed on untreated Dz-01-974, and the maximum HI (27%) was observed on Dz-01-196 inoculated by the PGPR consortium, which increases harvest index up to 169% over the control.

**TABLE 4:** Effect of PGPR inoculation on teff varieties yield and yield-related traits.

Treatment	SDW t/ha		GY t/ha		SY t/ha		HI%		LI%	
	M	D	M	D	M	D	M	D	M	D
Control	5.8^b^	5.9^b^	0.6^b^	1.22^b^	3.5^b^	3.7^b^	0.17^c^	0.16^b^	0.20^a^	0.21^a^
*S. marcescens ss marcescens*	14.0^a^	16.5^a^	0.9^a^	2.3^a^	7.9^a^	9.6^a^	0.25a	0.24^a^	0.25^a^	0.27^a^
*P. fluorescens biotype G*	13.8^a^	16.4^a^	1.7^b^	2.4^a^	7.4^a^	9.4^a^	0.22^b^	0.25^a^	0.26^a^	0.24^a^
*E. cloacae ss dissolvens*	13.7^a^	17.2^a^	1.6^b^	2.6^a^	7.6^a^	9.9^a^	0.20^b^	0.26^a^	0.24^a^	0.27^a^
PGPR consortium	13.8^a^	18.1^a^	2.0^a^	2.7^a^	7.7^a^	10.7^a^	0.27^a^	0.26^a^	0.25^a^	0.24^a^
LSD (5%)	1.02	0.2	0.2	0.15	0.2	0.88	0.08	0.04	2.63	2.57

M, magna; D, Dukem; SDW, shoot dry weight; GY, grain yield; SY, straw yield; HI, harvest index; LI, lodging index.

Different letters indicate significant differences at *P* ≤ 0.05 according to the LSD test.

### Effects of PGPR inoculation on teff grain nutrient uptake

The result of the ANOVA indicated that a significant difference (P ≤ 0.01) was observed in teff varieties on grain nitrogen (N), phosphorus (P), and calcium (Ca) uptake upon treatment (Table 5). Grain magnesium (Mg) and iron (Fe) uptake was significantly affected by teff varieties at a 5% probability level.

**TABLE 5:** Treatment and variety effects on teff grain nutrients uptake.

S.O.V	D.F	N%	P%	S	K%	Mg%	Ca%	Zn%	Fe%
TM	4	1.76**	1.88**	0.45*	0.006*^Ns^*	0.001*^Ns^*	0.06**	0.00001*^Ns^*	0.0002*^Ns^*
VT	1	0.01*^Ns^*	0.06^ Ns^	0.001*^Ns^*	0.003*^Ns^*	0.01*	0.001*^Ns^*	0.00002*^Ns^*	0.01*
Error	4	0.003	0.05	0.03	0.005	0.0004	0.001	0.00001	0.0003

N, nitrogen; P, phosphorus; S, sulfur; K, potassium; Mg, magnesium; Ca, calcium; Zn, zinc; Fe, iron.

*, **, and ***: statistically significant at *P* ≤ 0.05, *P* ≤ 0.01, and *P* ≤ 0.001 probability level, respectively; Ns, not significant; S.O.V, source of variation; D.F, degree of freedom; TM, treatment; and VT, variety.

#### Effects of PGPR inoculation on teff grain macro- and micronutrient uptake

Individual treatment means comparison showed that teff grain nitrogen (N%), phosphorus (P%), sulfur (S%), and calcium (Ca%) uptake was significantly affected by individual or consortium PGPR inoculation (Table 6). The maximum teff grain N (1.85%), P (3.35%), S (1.61%), and Ca (0.19%) uptake was observed on teff varieties inoculated with PGPR consortium, and the minimum teff grain N (1.42%), P (0.67%), S (0.38%), and Ca (0.06%) uptake was recorded on uninoculated control. Either individual or consortium PGPR inoculation did not significantly affect the uptake of potassium (K), Zinc (Zn), magnesium (Mg), and iron (Fe) although differences were recorded between the inoculated and uninoculated treatments.

**TABLE 6:** Individual treatment means PGPR inoculation on teff grain nutrient content improvement.

Treatment	N%	P%	S%	K%	Mg%	Ca%	Zn%	Fe%
Control	1.42^b^	0.67^c^	0.38^c^	0.44^a^	0.09^a^	0.06^b^	0.00^a^	0.04^a^
*Serratia marcescens ss marcescens*	1.78 ^a^	2.44^b^	1.28^ab^	0.36^a^	0.12^a^	0.07^b^	0.05^a^	0.05^a^
*Pseudomonas fluorescens biotype G*	1.76^a^	2.18^b^	1.41^ab^	0.42^a^	0.14^a^	0.08^a^	0.00^a^	0.04^a^
*Enterobacter cloacae ss dissolvens*	1.71^a^	2.07^b^	1.06b	0.31^a^	0.13^a^	0.09^a^	0.05^a^	0.04^a^
PGPR consortium	1.85 ^a^	3.35^a^	1.61a	0.44^a^	0.14^a^	0.19^a^	0.05^a^	0.05^a^
LSD (0.05)	0.17	0.60	0.40	0.17	0.09	0.09	0.006	0.09

N, nitrogen; P, phosphorus; S, sulfur; K, potassium; Mg, magnesium; Ca, calcium; Zn, zinc; Fe, iron.

Different letters indicate significant differences at *P* ≤ 0.05 according to the LSD test.

## Discussion

The present study showed that either single or consortia of PGPR inoculation had significantly improved the growth, yield, yield-related parameters, and grain nutrient uptake of the two teff varieties over the uninoculated plots. Similarly, Kumar et al. (2017) reported that inoculation of PGPR either alone or in various combinations significantly (*P* ≤ 0.05) increases the growth and yield of wheat compared with untreated controls.

The study indicated that the Dukem (Dz-01-974) variety responded better for a consortium inoculation for plant growth, yield, and related parameters as compared to the Magna variety. Zewdie et al. (2000) reported that higher GY responses were observed for the teff variety (Dz-01-096) compared with (Dz-01-354) by the inoculation of the Azospirillum isolates. Each teff variety responded differently to different bacterial inoculations indicating bacterial physiologic, metabolic, and root colonization ability differences, as well as the existence of some degree of specificity that might affect the growth, yield, and other parameters of the varieties.

plant height, not remove, panicle length, NOT remove, and the number of total spikelets are the most important traits that affect plant growth (Idota et al., 2015). In our study, teff height, plant height, and the number of total spikelets were significantly affected by inoculation of either single or consortium of PGPR. Woyessa and Assefa (2011) reported that inoculation of *P. fluorescens* and *Bacillus subtilis* significantly increased the growth of teff varieties. Longer PH and panicles allow more spikes that contain a better number of grains.

In this study, the SDW of both varieties was significantly increased by inoculation of either individual or consortium of PGPR inoculants. The maximum SDW was obtained from Dz-01-974 inoculated by the PGPR consortium. Meena et al. (2020) reported that the application of PGPR as a consortium of compatible strains has been more effective than their single application in the practical field. Similarly, Souza et al. (2015) reported that plant inoculation with a consortium of several bacterial strains might be an alternative to inoculation with individual strains, likely reflecting the various mechanisms employed by each strain within the consortium. PGPR consortium has a synergetic effect to mobilize essential nutrients, synthesizing different hormones and suitably beating the challenges like biotic and abiotic stress conditions as they had adapted to different environmental conditions. This could be due to an increase in the availability of essential soil nutrients and other substances through the synergistic effects of the PGPR consortium that can boost the SDW of the teff varieties. However, the lowest shoot weight was observed on uninoculated plots. This indicates that the experimental soils had limitations in releasing essential nutrients in adequate amounts to support teff crop shoot dry weight without any external addition of inputs.

There have been significant differences in teff GY by the inoculation of either single or consortium of PGPR. The highest GY was observed on Dz-01-974 inoculated with PGPR consortium, which increased 2.2 folds over control. Similarly, Woyessa and Assefa (2011) reported that teff varieties inoculated with native *P. fluorescens* and *B. subtilis* were significantly increasing GY by 28 and 44%, respectively. Sarma et al. (2009) reported that a mixture of two *P. fluorescens* strains increased *Vigna mungo* yield by 30% in comparison with the control. These could be for the reason that a consortium of PGPR increases the availability of the nutrients that are essential to enhance the yield of the plant by using various PGP mechanisms like phosphate solubilization, nitrogen fixation, and production of the different secondary metabolites, as well as improving plants tolerance to the biotic and abiotic stress factors.

The SY of cereal crops is an important agronomic parameter that is sensitive to soil nutrient availability or the nutrient applied from external sources (Tamene et al., 2017). In this study, the application of consortium PGPR inoculants significantly (*P* < 0.001) enhanced SY. Zafar-ul-Hye et al. (2020) reported that multi-strain inoculation with PGPR is more effective than single-strain inoculation to improve wheat (*Triticum aestivum*) SY. This could be due to the interaction effect of the bacterial consortium that improves the supply of unavailable nutrients and different hormones to the teff varieties.

Harvest index (HI) indicates the balance between the productive parts of the plant and the reserves. It indicates the presence of good partitioning of biological yield. In this study, the individual treatments’ mean result revealed that the harvest index of the teff varieties was significantly increased by the inoculation of consortium PGPR inoculants over control. These results showed that the PGPR consortium could improve the supply of essential nutrients to the plant and increase the harvest index.

No significant difference was observed in lodging index between the two varieties of teff upon inoculation by PGPR either alone or in a consortium although differences occurred between inoculated and uninoculated plots. PGPR inoculant might improve teff varieties’ stem strength by regulating the supply of nutrients and increasing root growth to prevent lodging problems.

Inoculation of either individual or consortium PGPR inoculants significantly improved grain N, P, S, and Ca uptake of the two teff varieties over control. Mantelin and Touraine (2004) reported that plants inoculated with PGPR significantly increased the uptake of nutrient elements like Ca, K, Fe, Cu, Mn, and Zn through proton pump ATPase. Moreover, Karlidag et al. (2007) reported that inoculation of *Bacillus* and *Microbacterium* inoculants improved the uptake of mineral elements by apple plants. Furthermore, Kumar et al. (2017) reported that co-inoculation of *Enterobacter* with *S. marcescens* and *M. arborescens* improved grain N and P uptake of wheat variety in the field experiment.

In general, this study confirmed that the PGPR consortium application was capable of enhancing the growth, yield, yield-related parameters, and grain nutrient uptake of the two varieties. However, the bacterial consortium displayed a marked difference in their effect on several features of growth and productivity of Dukem (Dz-01-974) teff variety. The variations perhaps originated by the PGPR consortium are differences in exerting PGP mechanisms and synergy to supplying essential nutrients to the teff varieties.

## Conclusion and recommendation

This study concluded that the utilization of native PGPR either alone or in a consortium as bioinoculants improves teff growth, yield, and grain nutrients uptake, as well as reduces the global dependence on hazardous chemical inputs, which threaten the environment, human health, and biodiversity. Furthermore, sustained teff production and productivity without affecting GY and quality of the grain nutrients is an important agricultural practice to meet consumer’s demand at the regional and national level. Further evaluation and demonstration could be conducted by inoculation of either individual or consortium PGPR inoculants on different crop varieties under different environmental conditions to explain the role of native PGPR as bacterial inoculants.

## Data availability statement

The original contributions presented in this study are included in the article/supplementary material, further inquiries can be directed to the corresponding author/s.

## Author contributions

ZT: conception and design of the work, data analysis and interpretation, and drafting the manuscript. ZT and FA: data collection. ZT, FA, AD, and TA: critical revision of the manuscript. FA, AD, and TA: final approval of the version to be published. All authors contributed to the article and approved the submitted version.
